# An *In vivo* Model for Short-Term Evaluation of the Implantation Effects of Biomolecules or Stem Cells in the Dental Pulp 

**DOI:** 10.2174/1874210600802010067

**Published:** 2008-04-29

**Authors:** Sally Lacerda-Pinheiro, Arnaud Marchadier, Patricio Donãs, Dominique Septier, Laurent Benhamou, Odile Kellermann, Michel Goldberg, Anne Poliard

**Affiliations:** 1 Laboratoire de Différenciation Cellulaire, Cellules Souches et Prions, IFR- 2937 CNRS, Villejuif, France; 2 Laboratoire de Caractérisation du Tissue Osseux par Imagerie, INSERM U658, Orléans, France; 3 Laboratoire Régénération et Réparation des Tissus Cranio-faciaux, EA2496, Faculté de Chirurgie Dentaire, Université Paris - Descartes, Montrouge, France

**Keywords:** Mouse incisor, dental pulp progenitor cells, amelogenin, experimental model

## Abstract

The continuously growing rodent incisor is a widely used model to investigate odontogenesis and mineralized tissue formation. This study focused on evaluating the mouse mandibular incisor as an experimental biological tool for analyzing *in vivo* the capacity of odontoblast-like progenitors or bioactive molecules to contribute to reparative dentinogenesis. We describe here a surgical procedure allowing direct access to the forming part of the incisor dental pulp Amelogenin peptide A+4 adsorbed on agarose beads, or dental pulp progenitor cells were implanted in the pulp following this procedure. After 10 days A+4 induced the formation of an osteodentin occluding almost the totality of the pulp compartment. Implantation of progenitor cells leads to formation of islets of osteodentin-like structures located centrally in the pulp. These pilot studies validate the incisor as an experimental model to test the capacity of progenitor cells or bioactive molecules to induce the formation of reparative dentin.

## INTRODUCTION

During dentinogenesis, pre-odontoblasts migrate from the central part of the pulp to the periphery of the embryonic pulp where the last cell division occurs [1]. When one of the daughter cells comes in contact with the basement membrane (BM) the cell becomes an odontoblast, whereas the other daughter cell located some distance away from the BM is incorporated in the Höehl’s cell layer [2]. Odontoblasts and Höehl’s cells constitute a specific subpopulation of neural crest-derived and paraxial derived mesenchymal-cells. After terminal differentiation they are responsible for dentin production (physiological and reactionary dentin). If the odontoblasts are injured or destroyed during a carious lesion or its treatment, Höehl’s cells may be reactivated and differentiate into new odontoblasts. Upon more severe injury, when the odontoblasts and Höehl’s sub-odontoblastic cell layer are irreversibly destroyed, dentin formation can be mediated by odontoblast-like cells recruited among pulpal cells during the process known as reparative dentinogenesis [3]. However, it is well known that the natural capacity for healing of a tooth is limited and that therapies involving calcium hydroxide are not always successful. Therefore the search for strategies stimulating or mimicking the natural healing properties of the dental pulp is important. Two types of such therapeutic strategies can be conceived today depending on the severity of the lesions. Firstly, implantation of bioactive molecules used as new pulp capping agents that would accelerate and/or induce the formation of a bridge of reparative dentin [4]. Secondly, implantation in an exposed pulp of purified stem/progenitor cells that would promote the formation of a reparative dentin.

Pulp implantation models have been developed over the years in different species (rat, rabbit, ferret [5-7], monkey [8, 9], dog [10], essentially to evaluate the long-term effects of bioactive molecules used as pulp capping agents. Mouse models have never been described, very likely due to their small size and hence difficulties in manipulating their teeth. However, the recent development of stem cell research, with all the data collected on mouse systems, makes it important to have access to a kin model.

The continuously erupting mandibular incisor has been extensively used to study the cellular and extracellular events involved in odontogenesis, because all the successive stages of development can be found in a single tooth, which exhibits many similarities with human tooth formation [11, 12]. A niche of stem cells was identified in the apical end region (cervical loop) of continuously growing teeth [13-15]. In the rat incisor, some of the stem cells continuously differentiate into odontoblasts, becoming functional, mature, old and finally degenerate [16]. Ever-growing teeth (hypselodont) such as the rodent incisors, maintain this function through a rich population of stem cells, whereas molars display limited growth (brachyodont) in rodents, and consequently have a reduced number of stem cells. Therefore, the murine incisors may provide a model to first dissect the cellular and molecular events underlying the regenerative processes.

In the present study, we have investigated whether the mouse mandibular incisor could be exploited as an experimental model for analyzing the potential of dental pulp progenitors cells or bioactive molecules to promote the formation of reparative dentin. A surgical technique was developed to gain an easy access to the apical forming end of the incisor by a limited perforation and to make a pulp exposure. An amelogenin peptide (A+4), which has been shown to act as a signaling molecule [17-19], and a dental pulp-derived odontoblast progenitor clone (17IA4) [17], were implanted into the pulp to validate the efficiency of the surgical approach. In both types of implantation, formation of a osteodentin-like neodentin was observed. Our data thus point to the validity of the mouse incisor as a tool to evaluate the potential of different types of stem cells or biomolecules for reparative dentinogenesis.

## MATERIALS AND METHODS

### Animals

Thirty lower incisors from 15 adults males C57Bl/6 mice (3 months old-25g) (Charles River - Lyon, FR) were used in these experiments. All experiments were performed under an institutionally approved protocol for animal research.

### Agarose Bead, Molecules and Cells Preparation

Affi-gel agarose beads (75-150µm in diameter, Biorad Labs, Hercules, CA) were used as a carrier for bioactive molecules and to facilitate the localization of the implantation sites. They were rinsed several times in PBS and distributed into a 12 microwell plate (10 beads/ wells) with or without (control beads) 2 µg of A+4. The plate was then incubated for 24h at 37°C to allow complete adsorption of the proteins at the surface of the beads.

17IA4, an odontoblast progenitor clone was derived from embryonic (ED18) pulp and cultured as described [20]. Approximately, 2.5 x 10^5^ cells combined or not with the agarose beads were distributed in Eppendorf tubes and centrifuged (1000 rpm / 1min) to form pellets which were thereafter implanted in the incisor.

### Surgical Procedure

**Fig. (1):**
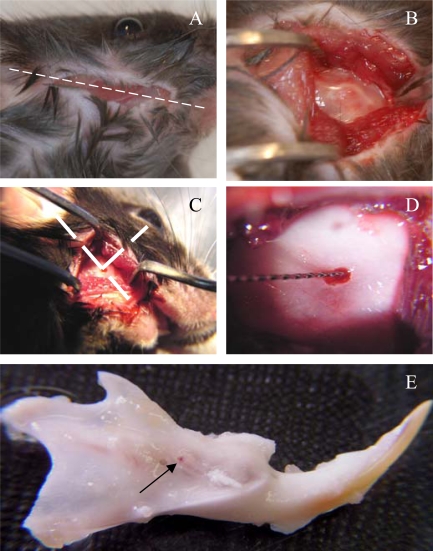
**Surgical procedure for implantation in the incisor pulp.**Incision was done along a theoretical line joining the auditory meatus and the lip commissure **(A)**, bone exposure **(B)** and perforation**(C)**. Cells or agarose beads were implanted **(D)** and the mandibles were dissected after 10 days **(E)**. Arrow points to the perforation site.

At day 0, the animals were anesthetized with an intraperitoneal injection of a solution of ketamine 20% (Imalgène^®^500, Bayer Pharma, Puteaux, Fr) and xylazine 5% (Rompun^®^2%, Merial, Lyon Fr) (10ml/kg). An incision of about 1cm long was made through the skin with fine scissors to access the subjacent muscle layer, along a theoretical line joining the auditory meatus and the lip commissure (Fig. **1A**). The masseter muscle was incised along its longitudinal axis with a scalpel blade. The periosteum was scrapped with a curette and the bone surface was exposed between the posterior angle of mandible and the molars block, approximately 1mm above of the mandibular basal border. The point where the outer oblique line crosses the apical loop of incisor was selected as a site for the pulp exposure (Fig. **1B,1C**). A low-speed dental drill equipped with a round tungsten-carbide burr size 6 (Dentisply-Maillefer, Ballaigues, Switzerland) was used to create a hole through the bone and tooth 9 (Fig. **1D**). The mice were divided into 5 groups of 3 animals each. They received group 1- agarose beads only, group 2- A+4 + agarose beads, group 3- 17IA4 cells + agarose beads (this group was performed as a control for cell implantation), group 4- 17IA4 cells alone, and group 5- sham (surgery without implantation). After implantation, the muscular layer was closed with one drop of cyanoacrylate and cutaneous layer was sutured with Vicryl-Monocryl* 4-0 (Ethicon – Jonhson&amp;Johnson, Piscataway, NJ, USA). All the animals received 10 mg/kg ketoprofen (Profenid, Paris, France) as analgesic immediately after surgery.

The mice were killed 10 days after implantation by cervical dislocation. Immediately after, the two hemi-mandibles were dissected out and fixed in a 4% paraformaldehyde solution overnight at 4°C (Fig. **1E**).

### Micro-Scanner Analyzes

The mandibles were scanned using a 1072 Skyscan® micro-CT (Skyscan®, Kontich, Belgium). The radiographic projections (n = 413) were acquired at 70 kV and 90 µA with a fixed exposure time of 4 seconds. Four frames were averaged for each rotation increment of 0.45° to increase the signal to noise ratio. The acquisition time was 2 hours. One thousand slices were reconstructed with the manufacturer reconstruction software (NRecon, Skyscan®) based on a modified Feldkamp algorithm. The resulting 3D dataset contained 1024 x 1024 x 1000 3.12 µm voxel elements. The 3D surface rendering of the mineralized tissues was made using the manufacturer visualization software (CtVol, Skyscan®) with a global threshold chosen halfway between grey level of soft tissues and hard tissues. The 3D grey level dataset and the 3D surface rendering allowed evaluating the opacity and the spatial distribution of the dentin formed.

### Histological Procedures

The samples were demineralized, in 4.13% EDTA solution. After dehydration in graded ethanol, the tissues were embedded in Paraplast plus (Kendall, Mansfield, USA). Sagittal (7 μm) sections were collected on SuperFrost slides (Menzel-Glaser^®^, Braunschweig, Germany). The slides were dewaxed, rehydrated and stained either with hematoxylin-eosin or Masson’s trichrome.

## RESULTS

**Fig. (2):**
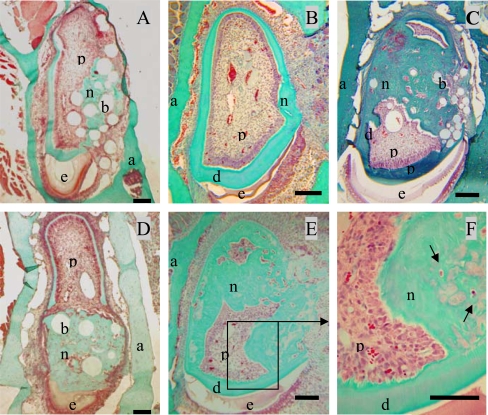
**Formation of neodentin ten days after implantation of the A+4 peptide or 17IA4 dental pulp progenitor cells in the pulp.**Control groups showed a limited formation of neodentin [11] around the beads **(A)** or occluding the pulp exposure ite **(B)**. Implantation of A+4 charged beads induced the formation of neodentin in the pulp compartment **(C)**. Implantations of the 17IA4 cells with **(D)** or without agarose beads **(E)** induced also the formation of osteodentin-like structures **(F)**. Pulp (p), dentin (d), enamel (e), alveolar bone [29].Bar 200µm.

**Fig. (3):**
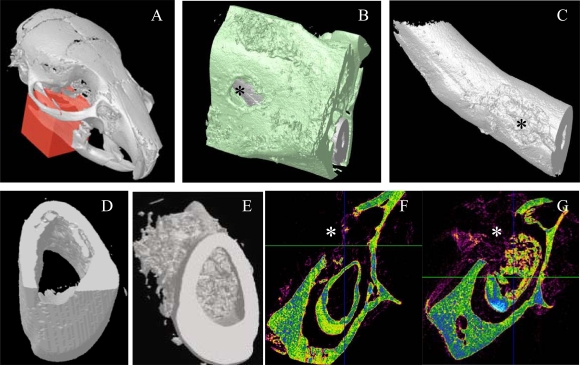
**Micro-scanner analyzes of incisor implanted with the 17IA4 cells.** The red block indicates the zone analyzed **(A)** and the asterisk(^*^) corresponds to the perforation site in the bone **(B)**, and in the tooth **(C)**. Ten days after implantation in the pulp, no mineralization was observed in the control group **(D,F)** in contrast, the 17IA4 group formed a large mineralized structure within the pulp **(E,G)**.

Ten days after implantation, no inflammatory process, infection or tissue necrosis was detectable in the treated mandibles, and despite the surgery, the soft tissues were involved in a healing process. 17/18 incisors were implanted successfully. In the two control groups 1 and 5, implanted with agarose beads alone or after surgical preparation without any implantation, respectively reparative dentin formation was limited to area located around the beads or occluding the pulp exposure site (Fig. **2A**, **2B**). Implantation with A+4-soaked agarose beads (group 2) led to the formation of a diffuse mineralized structure within the pulp as evidenced by histological (Fig. **2C**) and microscanner analysis (data not shown). The newly formed tissue is reminiscent of osteodentin, since the cells were trapped into osteoblast-like structure. Implantation of the progenitors cells (17IA4), alone or in association with agarose beads (groups 3 and 4), also promoted, in both cases, the formation of islets of osteodentin-like structures in the pulp (Fig. **2D-F**). To assess the degree of mineralization of the newly formed dentin, 3D analyses by X-ray microscanner of the hemimandibles were performed (Fig. **3A-C**). The pulps of the control and sham groups were radiolucent (Fig. **3D,3F**). In contrast, those implanted with the progenitor cells (Fig. **3E,3G**) or the bioactive molecules (data not shown) displayed a radiopacity very similar to dentin or bone. The microscanner analysis allowed determining that 10 days after implantation, the zone of mineralized neodentin has extended within the pulpal compartment over a distance of roughly 1milimeters.

## DISCUSSION

### Experimental Approach

We have developed an **in vivo** experimental approach that allows a direct access to the pulp of the forming mouse incisor. As no vital organ is concerned, the animals recover promptly from the surgery and it is possible to evaluate the biological effects of implantations of cells or molecules over a period of 1 to10 days. Afterwards, endogenous aging processes, and odontoblast and pulp degeneration [16, 21], may interfere with the biological reaction under investigation. Previous studies have used a similar surgical approach by drilling a cavity through the basal bone to gain access to the enamel organ of the rat incisor [22, 23]. Another surgical attempt to selectively target the odontogenic organ was described, using an osmotic minipump connected to a bony window overlying the apical end of the rat incisor [24]. These approaches were mainly aimed at studying the effect of pharmacologic agents on the formation of dental mineralized tissues. Our strategy was focused on gaining easy access to the dental pulp, without perturbation of the enamel organ and of the stem cells niche located at the apex of the incisor [13, 25]. The present results establish our method as a useful and reliable approach in evaluating the capability of progenitors cells or bioactive molecules of promoting dentin regeneration or repair.

### The Control Groups 

The rodent incisors as other pulpal implantation models, present by itself a capacity of self-repair [6, 7, 26, 27]. Scaffolds of gelatin, collagen, alginate or agarose beads have been used as carriers for molecules implanted in the exposed pulps and may not be completely biological [9, 28]. The control groups did not display any reparative mineralization in the pulp, and showed only a limited amount of reparative dentin at the exposure site as expected.

### The Effects of the A+4 Implantations

The small molecular weight amelogenin gene spice product (A+4) has been identified as a molecule expressed by the odontoblasts. It appears to function as a signaling molecule [18, 19]. A+4 had been shown to promote chondrogenesis and osteogenesis either **in vitro** or when implanted in ectopic sites [19, 29, 30]. When used as capping-agent in rat molars, A+4 induce the formation of a true dentinal bridge, occluding the pulp exposure in 15 to 30 days [31]. After implantation in the pulp of molars, A+4 stimulates the recruitment (commitment) of cells that proliferate and differentiate into osteo/odontoblast-like progenitors, which after terminal differentiation will form a reparative osteodentin-like structure. The bioactive potential of A+4 was confirmed in the present study in the incisor model since formation of a significant amount of neodentin is promoted by this peptide in the dental pulp. In this context, as a continuously growing tooth containing an identified stem cell niche , the incisor may constitute a sensitive tool, to study the early stages of the dentinal repair process promoted by the amelogenin peptide, namely the recruitment steps of the pulp progenitors.

### The Effects of the Progenitors Cells Implantations

Stem cells have been isolated and characterized in the human dental pulp. Significant effort is devoted to determine their therapeutic potential. After ectopic transplantation (sublingual region, subcutaneous), they produced a bone or dentin matrix [32-34]. However, up to now, no report provides data on the ability of these cells to directly contribute to reparative dentin formation after direct implantation into an injured dental pulp. The experimental approach used here provides some hints that such would be the case. Indeed, implantation of the clonal progenitor cells, 17IA4, into the pulp leads to the production of a large amount of reparative-like dentin. In contrast to the diffuse dentin formed after A+4 implantation, the 17IA4 cells promote the formation of a dentin mostly located in the central zone of the pulp. Microscanner analysis confirms the histological data on neodentin formation further demonstrating the mineralization process and its extent within the pulpal compartment. The formation of neodentin in the pulp may be explained by either the 17IA4 cells differentiating toward an odonto/osteoblast phenotype and forming a osteodentin-like structure, or by these cells producing signals that stimulate the commitment, proliferation and differentiation of some resident progenitors cells. These alternative hypotheses are now under evaluation and preliminary results suggest that the implanted progenitor cells are indeed capable of producing neodentin (data unpublished).

In conclusion, the data presented herein validate the use of the mouse incisor model as a tool to obtain a better understanding of the mechanisms of reparative dentin formation stimulated by biomolecules or cell implantations. Therefore, it should also allow the study of the efficiency of new therapeutic agents that may be used as pulp capping agents. Finally, our results of cell implantation in the pulp show the capacity of dental pulp progenitors of contributing to dentin formation in an injured tooth, paving the way for initiating studies on cell therapy approaches of dentin injuries.
